# A Framework for Improving the Quality of Research in the Biological Sciences

**DOI:** 10.1128/mBio.01256-16

**Published:** 2016-08-30

**Authors:** Arturo Casadevall, Lee M. Ellis, Erika W. Davies, Margaret McFall-Ngai, Ferric C. Fang

**Affiliations:** a Department of Molecular Microbiology and Immunology, Johns Hopkins Bloomberg School of Public Health, Baltimore, Maryland, USA; b Division of Surgery, Department of Surgical Oncology, University of Texas MD Anderson Cancer Center, Houston, Texas, USA; c American Society for Microbiology, Washington, DC, USA; d Pacific Biosciences Research Center, University of Hawaii at Manoa, Honolulu, Hawaii, USA; e University of Washington School of Medicine, Seattle, Washington, USA

## Abstract

The American Academy of Microbiology convened a colloquium to discuss problems in the biological sciences, with emphasis on identifying mechanisms to improve the quality of research. Participants from various disciplines made six recommendations: (i) design rigorous and comprehensive evaluation criteria to recognize and reward high-quality scientific research; (ii) require universal training in good scientific practices, appropriate statistical usage, and responsible research practices for scientists at all levels, with training content regularly updated and presented by qualified scientists; (iii) establish open data at the timing of publication as the standard operating procedure throughout the scientific enterprise; (iv) encourage scientific journals to publish negative data that meet methodologic standards of quality; (v) agree upon common criteria among scientific journals for retraction of published papers, to provide consistency and transparency; and (vi) strengthen research integrity oversight and training. These recommendations constitute an actionable framework that, in combination, could improve the quality of biological research.

## EDITORIAL

In the second decade of the 21st century, investigators in the biological sciences are making tremendous progress in a wide variety of fields. Despite great progress, and with optimism for even greater discoveries in the future, there is also a sense of crisis as numerous indicators suggest that there are systematic problems with the quality and reliability of research in the field. These indicators of concern include a marked increase in the number of retracted papers, with most retractions being caused by some form of misconduct (1); reports of low reproducibility for published studies (2–4); surveys showing a high prevalence of questionable research practices among scientists (5); and the finding that a disturbingly high number of papers contain an inappropriately manipulated image (6). Some have suggested that the poor quality of science is contributing to a slowing in therapeutic innovation in the biomedical sciences (7, 8). In this environment, the American Academy of Microbiology (AAM) convened a 2-day colloquium in the fall of 2015 entitled “Promoting Responsible Scientific Research” that explored the problems plaguing the biological sciences and provided six recommendations in the hope of creating a framework for improving the quality of research (http://academy.asm.org/index.php/browse-all-reports/5512-promoting-responsible-scientific-research). In this essay, we summarize the major findings and recommendations from the colloquium.

Colloquium participants agreed that there is a problem of reproducibility in the biological literature, although there was also a recognition that even the term “reproducibility” can be problematic since it can mean different things to different people (9). For example, a recent survey of ~1,500 scientists found considerable confusion on what is meant by reproducibility and wide divergence of opinion as to its causes (10). The colloquium participants identified the three major causes of lack of reproducibility as (i) sloppy science, (ii) selection and experimental bias, and (iii) misconduct, with general agreement that misconduct gathers the most attention but is likely to be the least important contributor to the overall problem. There was consensus that systematic problems in the conduct of science are likely to be responsible for most instances of irreproducible research, including laboratory errors as documented in honest retractions not due to misconduct (11), lack of or inappropriate controls, faulty statistical analysis, invalid reagents such as contaminated cell lines, favoring certain experimental outcomes over others, disregard of contradictory data, and bias in data selection and use. Colloquium participants agreed that piecemeal fixes to science were unlikely to have a major effect on the quality of biological research and suggested six recommendations as part of a comprehensive effort to improve the enterprise.

## DESIGN RIGOROUS AND COMPREHENSIVE EVALUATION CRITERIA TO RECOGNIZE AND REWARD HIGH-QUALITY SCIENTIFIC RESEARCH

The current preoccupation of many scientists with publishing their work in a journal with the highest journal impact factor (JIF) is having a detrimental effect on the biological sciences. Despite almost universal condemnation of the use of JIF to assess the significance of scientific work, the use of the JIF in rating publications and scientists remains highly prevalent in decisions involving hiring, funding, and promotion (12). The relentless pursuit of high-IF journals as publishing venues has been given clinical names such as “journal mania” (13), “IF mania” (12), and “impactitis” (14), and among its consequences is “impacted science” (15). JIF mania bears on the reproducibility problem because it produces intense pressure on scientists to publish in journals with the highest IF, and these journals often require clean stories that could lead some authors to sanitize their data or overstate the conclusions to make their papers more attractive in a process that runs the gamut from bad science to outright misconduct. In this regard, there is a positive correlation between the retraction index of a journal and its impact factor (16). Given these concerns with the misuse of the JIF, the ASM Journals have removed JIF information from their journal websites as a statement of principle (17).

Among participants, there was resignation to the fact that we are living at a time of rankings, as evident from the widespread use of numerical ratings when evaluating everything from the quality of colleges to wine. Given this environment, it is inevitable that some form of metric will be applied to science. Scientists are already judged on their H-index, which is an author-level metric based on the number of publications and their citations (18). However, no validated index exists for evaluating the quality of scientific work. Although the importance of scientific work can be very difficult to ascertain at the time of publication (19), there was discussion that it is theoretically possible to develop a metric that evaluates the quality of a published paper. Such a metric could include the appropriateness of statistical analysis, documented replications, and quality of the methods, validation, or reagents, etc., criteria which are already used by individual scientists when they evaluate publications in their field. Whereas such a metric would necessarily involve some degree of judgment, it was perceived that the development of a quality indicator is a promising area for future research in the metrics of science. In this regard, it is worthwhile to note recent efforts to develop other metrics such as the relative citation ratio (20), which aims to generate an estimate of the influence of a publication in a particular field. Although the relative citation ratio is a vast improvement over the use of JIF to gauge the importance of a publication, it still relies on citations and thus cannot be used to evaluate scientific quality in real time.

## REQUIRE UNIVERSAL TRAINING IN GOOD SCIENTIFIC PRACTICES, APPROPRIATE STATISTICAL USAGE, AND RESPONSIBLE RESEARCH PRACTICES FOR SCIENTISTS AT ALL LEVELS, WITH TRAINING CONTENT REGULARLY UPDATED AND PRESENTED BY QUALIFIED SCIENTISTS

Given that the quality of a scientist’s output is often a reflection of his/her training, one obvious mechanism to improve the quality of biological research is to improve the training of scientists. Graduate training programs leading to Ph.D. degrees in the biological sciences require completion of a set of didactic courses in the chosen area of study plus original research that is organized into a thesis. Biological data are increasingly numerical and amenable to analysis with mathematical tools. However, current graduate programs vary significantly with regard to their requirements for mastering probability and statistics, despite the widespread use of statistical tests in the analysis of data. Today, most statistical analysis is done with programs that produce a result, most often a *P* value or some measure of correlation, without requiring foundational knowledge of the statistical analyses involved. The combination of an overreliance on *P* values with the lack of a full understanding of what is meant by testing the null hypothesis has led to the misuse of the statistics, such that the American Statistical Association has issued a warning on the use of *P* values, and at least one journal has banned their inclusion in research articles (21–24). Misuse or even abuse of statistical analyses can lead to assertions that are not true, which may be contributing to the problem of reproducibility. This problem can be addressed by including formal statistical training in the graduate curriculum and providing regular refresher courses as a form of continuing scientific education. More complex studies may require real-time input and collaboration from statisticians (including informaticians).

In addition to formal statistical training, the participants agreed that there is a need for more formal training in proper experimental design. Currently, students learn experimental design from their mentors, who may not be well versed in good experimental design, or from the literature, which may provide bad examples. For example, the use of positive and negative controls, the determination of dose-response relationships and time courses, awareness of instrumental and experimental errors, the demonstration of phenomena by multiple methods, and the systematic perturbation of experimental variables to test predictions are each fundamental aspects of robust experimental design. Remarkably, most graduate programs do not have didactic mechanisms to teach this knowledge and instead rely on mentors. There was consensus from the participants that such courses should be developed and taught as part of the curriculum for students/fellows/trainees in investigative careers. Statistical training can improve reasoning, and the combination of formal training in best scientific practices and that in statistics could produce synergistic effects (25). Although some expressed the concern that additional coursework could prolong graduate education, there was also the counterview that much of the time during the research phase of the Ph.D. years/postdoctoral fellowship (for those not previously in a Ph.D. program) is poorly used, and better preparation in experimental design could shorten the time to graduation by increasing the quality of the data produced.

Improving the quality of biological science research could also be aided by the development of a best practices concept for each field. For example, in the experimental preclinical medical sciences there is concern that some of the lack of reproducibility is caused by reagent problems such as contaminated cell lines (26), antibodies with poor specificity (27), and poor standardization of protein reagents (28). In fact, when it comes to using antibodies in research, nearly a third of investigators do not validate antibodies and instead expect them to work as advertised (29). Greater attention to authenticating the quality of reagents used in research could improve the quality of results and thus enhance the likelihood that the results are reproducible.

## ESTABLISH OPEN DATA AS THE STANDARD OPERATING PROCEDURE THROUGHOUT THE SCIENTIFIC ENTERPRISE

The principle of open data is that all of the data that are used, generated, and analyzed in a scientific study should be accessible to interested parties. Establishing open data as a standard operating procedure can enhance the quality of biological research, since the inspection of primary data may reveal causes for irreproducibility. In recent years, the biological sciences have entered the era of “big data” as exemplified by the increasing use of large “omics” data sets and population studies. The results of big data studies are often highly dependent on how the data are analyzed. Differences in the ways that investigators analyze data can lead to major differences in results or conclusions, which can contribute to the reproducibility problem in biology. Making primary data available to all interested parties could allow other investigators to validate primary conclusions as well as identify sources of discordance when study results differ.

The benefits of open data would also apply to routine laboratory experimental research. Journals seldom publish an entire primary data set, and what is published is usually presented in the form of graphs and figures that have processed the primary data. Furthermore, investigators tend to publish the results that best fit the conclusions of the study, and information about the replicability of experiments may not be complete. For example, a statement that an experiment was replicated three times is true even when the experiment yielded the described outcome only some of the time. Compliance with the open data principle is likely to require major changes to the laboratory culture that could include mandatory use of electronic laboratory notebooks using platforms that are compatible across laboratories. Simple measures such as recognizing authors who comply with open data policies can result in large increases in participation, suggesting the power of positive incentives in making data more available (30). In general, open data policies should begin at the time of publication. In this regard, we note the example and precedent used by the National Cancer Institute in data sharing as part of the effort to hasten the development of new cancer cures (http://www.cancer.gov/news-events/cancer-currents-blog/2016/datapalooza-moonshot).

## ENCOURAGE SCIENTIFIC JOURNALS TO PUBLISH NEGATIVE DATA THAT MEET STANDARDS OF QUALITY

The scientific literature is highly biased toward publishing positive results. This is true in multiple disciplines and includes clinical trials in which negative study results are often not published or are delayed compared to publication of positive studies (31–33). The bias toward positive results is easy to understand since a negative result may reflect inadequacies in the study (false negative) or a true negative. Given this uncertainty and the fact that proving a negative conclusion is not possible, investigators, journals, and reviewers tend to be more interested in positive results. The bias toward publishing positive studies plays into the fact that these studies may be practice-altering, with a high number of citations, increasing the JIF and the reputation of the investigators. In addition, the influence of industry on the selective publication of positive results cannot be ignored (including the hiring or financial support of medical writers), and there is little to be gained by sponsoring companies in regard to publishing negative studies. The bias toward positive results combined with the limitations of experimental design has led to the controversial and provocative suggestion that most research findings are false (34).

The colloquium participants agreed that there is a need for the publication of negative results of studies that meet the standards for research quality in individual fields. Given the uncertainties inherent in negative studies mentioned above, there was agreement that such studies may need to go further in improving experimental design to amass convincing evidence that a result is indeed negative. Publishing more negative data may also require a change in journal practices or the creation of specialized publication venues for such studies. There was consensus that well-done studies that produce negative results should be published, and the availability of those results could improve the literature by revealing positive findings that are not reproducible. Furthermore, colloquium participants welcomed the validation initiatives that aim to establish the reproducibility of key studies.

## AGREE UPON COMMON CRITERIA AMONG SCIENTIFIC JOURNALS FOR RETRACTION OF PUBLISHED PAPERS, TO PROVIDE CONSISTENCY AND TRANSPARENCY

Retractions of published papers provide a mechanism for correcting the literature by identifying work that is no longer considered to be valid. Retractions may be regarded as falling into two general categories: honest and dishonest (16). Honest retractions are those that result from honest mistakes in the research, such as error, methodological inadequacies, and/or reproducibility, while dishonest retractions are the result of misconduct, such as plagiarism or fabrication/falsification of data. Most retractions fall into the dishonest category and result from some form of misconduct (1). Retractions are usually announced by the journal that published the original paper in the form of a retraction notice, which is typically a short note that is electronically linked to the original publication to warn readers that the research is not valid. Retraction notices vary greatly in their information content explaining the causes for the retraction. Some retraction notices provide detailed information on what led to the withdrawal of the study, whereas others provide little or no information. For example, until 2015 the *Journal of Biological Chemistry* provided no information on the causes for retraction in their retraction notices, a policy that has now changed (35). Adding to the problem of informational content in retraction notices is the fact that retraction notices are often incomplete or misleading and may attribute the cause for retraction to laboratory problems that are subsequently found to be misconduct (1).

Colloquium participants agreed that retraction notices are an essential ingredient for preserving the integrity of the literature and called upon scientific organizations and journals to develop a set of common criteria that ensure consistency and transparency in announcing the causes for retraction, including open access (i.e., access free of charge) to retraction notices. Retraction notices provide an important window into the mechanisms by which the process of scientific research can go astray, and these can be informative in identifying strategies to reduce error. For example, an analysis of causes for retractions due to errors, not misconduct, identified common sources of error that can be used to develop best practices to improve the quality of science (11). Like accident investigations that seek to identify correctable causes and thus reduce the likelihood of future accidents, a robust and informative process for reporting the causes of retraction could provide information to minimize future errors.

## STRENGTHEN RESEARCH INTEGRITY OVERSIGHT AND TRAINING

Misconduct in science has devastating professional consequences for those who commit it (36). A finding of misconduct is a career-ending event for most scientists, as evidenced by dramatic reductions in subsequent productivity and the ability to obtain research funding (36). Misconduct in scientific studies with clinical implications can result in direct harm to patients and affect social attitudes toward public health recommendations. For example, fraudulent and now retracted data showing an association between measles vaccination and autism have contributed to skepticism about vaccine safety that has in turn translated into lower vaccination rates and a resurgence of measles outbreaks (37, 38).

Training in ethics and the responsible conduct of science is already a common feature of scientific training programs. However, this training is often seen more as a rite of passage to be completed in the quest for a scientific degree than as an integral component of a system that seeks to improve the quality of science. Current training in research integrity is largely focused on young scientists who are in educational programs and is accomplished in the form of didactic courses or case studies that seek to teach ethical principles. However, an analysis of scientists found to have committed misconduct shows that the problem is prevalent throughout all ranks, ranging from students to established investigators (39). This finding suggests the need to increase the focus of research oversight and training to all members of the research community irrespective of their academic rank. In addition, we must educate trainees and faculty on actions to take if misconduct is suspected. This may be dependent on the country or institute, but “whistleblowers” must have a safe environment to bring suspected misconduct to the attention of universities and funding agencies.

## SUMMARY

We are aware that some of these recommendations echo those made by other authorities, and by restating them, we affirm them. We recognize that that these recommendations do not include all the facets of science that require attention and improvement, and we use the word framework to highlight the notion that these recommendations can be built upon by additional recommendations as more information becomes available regarding problems and solutions. At a time when society is beginning to reap the rewards of the revolution in molecular biology, there is great urgency for the biological sciences to clean up their act so that this research can continue to find solutions to problems facing humanity ranging from the threat of epidemics to the failing green revolution and climate change. In this regard, the six recommendations made by the colloquium participants provide an actionable framework to improve the quality of biological research. In addition, it will be essential to address structural issues in the contemporary scientific enterprise that are contributing to poor research practices by creating excessive competition among scientists for employment opportunities and funding (40).
